# Investigation of Thermal Behaviour of a Hybrid Precasted Concrete Floor using Embedded Sensors

**DOI:** 10.1186/s40069-018-0287-y

**Published:** 2018-10-05

**Authors:** Shane Newell, Jamie Goggins

**Affiliations:** 1 Civil Engineering, School of Engineering, Alice Perry Engineering Building, National University of Ireland Galway, Upper Newcastle, Galway, Ireland; 2 Department of Building and Civil Engineering, Galway-Mayo Institute of Technology, Galway, Ireland; 3 Ryan Institute for Environmental, Marine and Energy Research, NUI Galway, Galway, Ireland; 4 Centre for Marine and Renewable Energy Ireland (MaREI) Research, Galway, Ireland

**Keywords:** thermal behaviour, hybrid precast concrete floor, vibrating wire gauges, real-time monitoring, environmental performance, structural health monitoring

## Abstract

Concrete structures expand and contract in response to temperature changes which can result in structural strain and cracking. However, there is a limited amount of robust field data on hybrid concrete floor structures. Shortage of such data impacts on our understanding of how concrete structures respond to thermal effects and ultimately the overall design of concrete structures. Thus, a comprehensive structural and environmental monitoring strategy was implemented by the authors during the construction of an educational building. Sensors were embedded in the precast and in situ components of a hybrid concrete lattice girder flat slab so that the thermal response of the floor during the manufacture, construction and operational stages could be investigated. Many aspects of the thermal behaviour of the floor during the construction phase were monitored using the embedded sensors. The early-age thermal effects during curing and the impact of the variation of ambient temperature (daily and seasonal) and solar radiation on the behaviour of concrete floor is explored in the paper. Values for restraint factors and the in situ restrained coefficient of thermal expansion of concrete are calculated using the data from the embedded sensors. Numerical modelling of the thermal behaviour of the hybrid concrete floor was undertaken and validated using the real-time field measurements. The data presented and analysed in this paper can be used to improve the understanding and modelling of the thermal behaviour of a hybrid concrete floor. This will assist with improved design of sustainable buildings as it allows the environmental performance of the floor to be optimised with respect to controlling the internal environment, thermal mass and energy efficiency.

## Background

Most concrete structures are subjected to frequent temperature changes during their design life. In fact, the internal temperature of a reinforced concrete element and relative humidity can be considered as two of the key indicators to monitor and evaluate the condition of the material and detect structure deterioration (Xia et al. 2006; Duffó and Farina 2009; Yuen and Kuok 2010). For example, Hover (2010) explored the similarity between concrete and the human body in regard to the influence of temperature and moisture conditions on health and performance. The expansion and contraction of concrete structures in response to temperature can result in structural strain and cracking. The strain through a winter/summer cycle can be up to ten times greater than that due to service loading (Buenfeld et al. 2008). Furthermore, consideration of thermal effects is particularly important for designers checking the possibility of early-age thermal cracking. Movement of concrete elements due to thermal effects is unavoidable due to the heat of hydration as the concrete hardens. During the hydration process, the concrete expands as the heat of hydration exceeds the rate at which heat is dissipated, but then the concrete contracts as the concrete cools down to ambient temperature. These volume changes would be of little consequence if the concrete was unrestrained and free to expand and contract without creating any stresses. However, in real structures, most concrete elements have some form of restraint (internal or external) and, therefore, stresses are generated which have the potential to cause cracking which can affect the long-term performance and durability of concrete structures. Hence, it is very important to understand the thermal response of concrete components (concrete temperature, thermal strain etc.) so that better prediction models may be developed.

Knowledge of the thermal behaviour of concrete components can also be used to improve the energy performance of buildings and the thermal comfort of the occupants (Hajdukiewicz et al. 2015). For example, Hoseggen et al. (2009) found that exposed concrete in the ceiling of an office building on the Nord-Trøndelag College (HiNT) campus in Norway both considerably reduces the number of hours with excessive temperatures and created a better and more stable thermal environment during the working day when compared to utilising a suspended ceiling.

Many modern buildings have a building management system (BMS) that monitors and controls the environmental conditions and energy consumption during the operational phase of the building life cycle. The use of embedded sensors to monitor the actual behaviour of components within the building complements the data from the BMS and allows a more holistic evaluation of the performance of the building. Linking data from embedded temperature sensors in the concrete floor slabs with the room air temperature and external weather conditions in the BMS could assist with making the most effective use of thermal mass within a building in controlling internal comfort conditions and minimising energy consumption. The additional advantage of the embedded sensors is that they can be used to study the behaviour of components at all stages of its life cycle (manufacture, construction, operation).

Ge et al. (2014) conducted an experimental study investigating the accuracy of various embedded strain sensors (vibrating-wire strain gauge, electrical resistance gauge, Fibre Bragg grating sensor and Brillouin optical fibre sensor) to measure the response of concrete beams subjected to thermal loading. They concluded that vibrating-wire strain gauges produced the most stable and reliable results (compared to theoretical calculations) among the four sensors. In addition, following an experimental and numerical study of VW strain gauges with metallic and plastic housing, Azenha et al. (2009) recommended the use of metallic VW strain gauges for early-age concrete strains as they are more robust than those that use plastic housing and present lower temperature sensitivity.

**Fig. 1:**
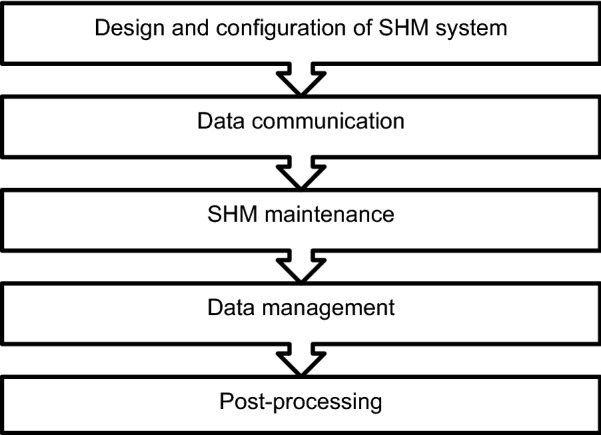
Visual schematic of the SHM methodology (Newell et al. 2016b).

Observations and findings from embedded instrumentation in a hybrid precast concrete flat slab floor in a recently constructed educational building are presented in this paper. The embedded sensors permitted real-time monitoring of the floor during the manufacture, construction and operational phases of the building. The embedded sensors in this project are part of a structural health monitoring (SHM) strategy which has been developed in the National University of Ireland Galway (NUI Galway) to monitor the structural and environmental performance of a number of educational buildings (Newell et al. 2016a, b; Hajdukiewicz et al. 2015). SHM can be defined as a non-destructive in situ structural evaluation method that uses any of several types of sensors which are attached to, or embedded in, a structure (ISIS Canada 2005). A schematic summarising the SHM methodology which was implemented for this project is shown in Fig. 1. The data from the sensors can be used to assess the safety, integrity, strength, or performance of the structure, and to identify damage at its onset.

In this paper, details are provided of a case study building, floor structure and the extensive instrumentation installed in the floor. Monitoring of the floor using the embedded sensors is ongoing with automatic data logging at frequent time intervals. This paper presents the results for the first 12 months of the building prior to the operational phase of the building. The results show the transient, seasonal and diurnal thermal response of the concrete floor.

## Methods

### Case Study Building

**Fig. 2:**
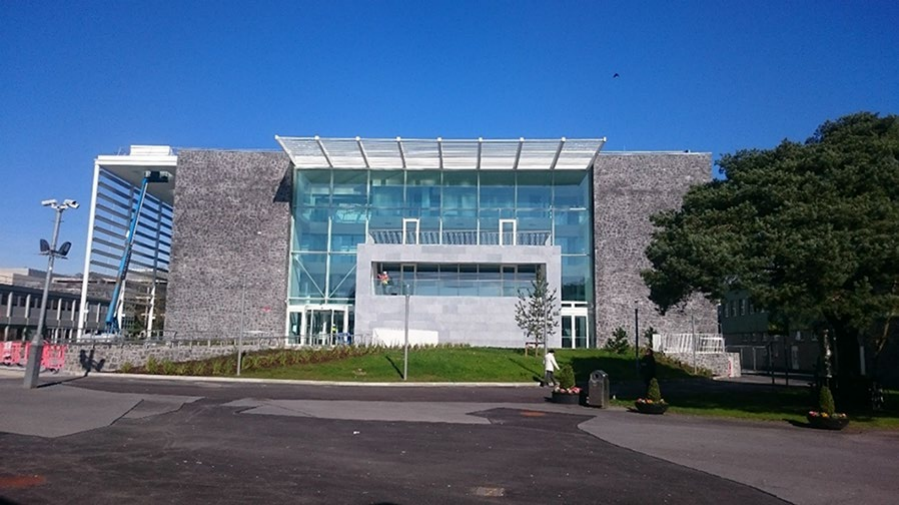
Human biology building.

The Human Biology Building (HBB) on the NUI Galway campus is the new home to three existing schools: Anatomy, Physiology & Pharmacology and Therapeutics (Fig. 2). The building is a teaching and research facility for undergraduate and postgraduate students and incorporates laboratories, lecture theatres and meeting rooms. The building is a five storey building including basement and two storey plant enclosure at roof level. The building has a gross floor area of 8200 m^2^ and the project cost is in excess of €30 million. Construction commenced in January 2015 and the construction period was 22 months.

The basement was constructed using in situ concrete and above ground the majority of the structure was constructed using precast concrete components such as twinwall system, hybrid concrete lattice girder slabs and hollowcore slabs. All precast components were designed, manufactured and installed by Oran Pre-Cast Ltd., who is located 10 km from the site.

**Fig. 3:**
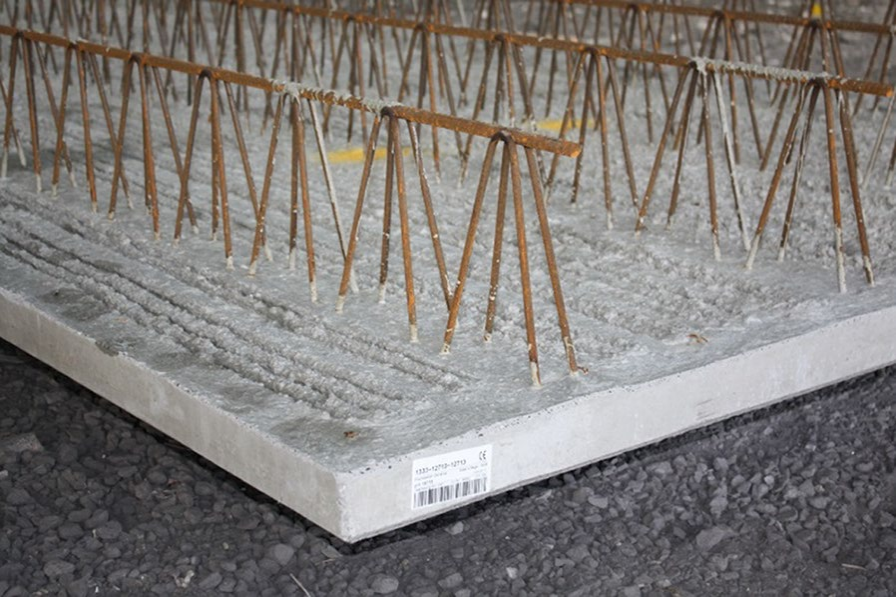
Precast concrete lattice girder plank.

The majority of the floor structure in the HBB was constructed using two-way spanning hybrid concrete lattice girder flat slab floor system (either 400 or 250 mm thick). This floor system was selected by the contractor to reduce construction time, reduce steel fixing on site, minimise formwork, improved safety and because of its high quality finish. The lattice girder plank (Fig. 3) consists of a 65 mm thick concrete slab, which contains the bottom layer of reinforcement of the slab and a steel lattice girder which protrudes from the plank. The lattice girder planks are temporarily propped when erected on site and the top layer of reinforcement is placed on site prior to pouring the structural concrete topping. Steel reinforcement ‘stitching’ bars are also required to be placed across adjacent precast planks to compensate for the discontinuity caused by discrete planks, whose size is limited by transportation logistics. Thus, two-way spanning action is achieved by positioning these ‘stitching’ bars across the joints between adjacent planks. The steel lattice girder is required to provide stiffness to the plank during construction and also to improve the composite action between the plank and structural topping. The props are removed when the structural topping has reached the required compressive strength.

### Embedded Sensors and Data Acquisition

**Fig. 4:**
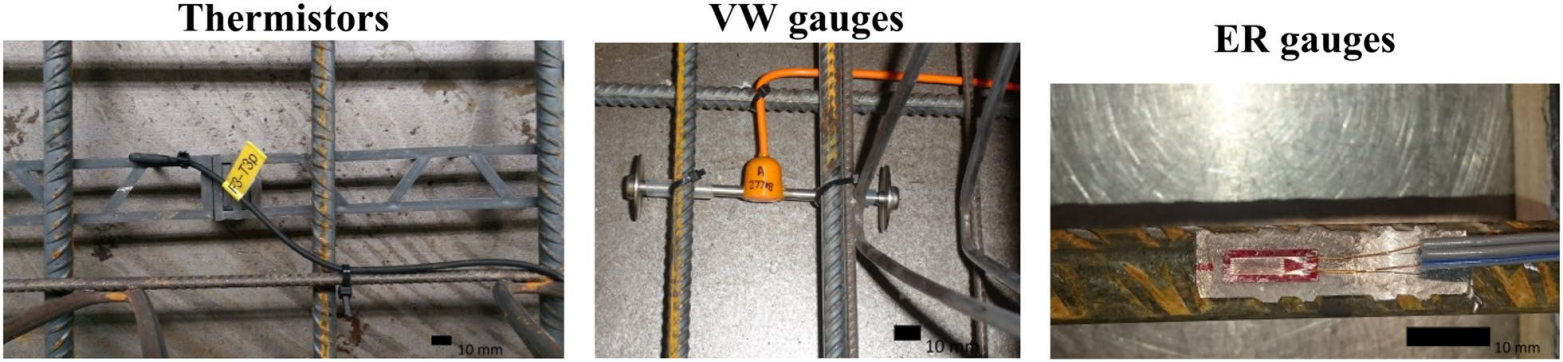
Sensors embedded in the buildings’ structure (Newell and Goggins 2017).

This project used a number of sensors (Fig. 4) which were embedded in the floor structure to monitor the environmental and structural performance of the hybrid concrete lattice girder flat slab:

- Vibrating Wire (VW) gauges (Gage Technique model TES/5.5/T) embedded in concrete that measure strain and temperature. The range of the measured strains is greater than 3000 microstrains and the resolution is better than 1 microstrain. The measured temperature range is − 20 to + 80 °C with an accuracy of ± 1 °C.
- Electrical resistance (ER) strain gauges (Tokyo Sokki Kenkyujo model FLA-6-11) bonded to steel reinforcement in concrete, which measure uniaxial strain. The ER gauges have a strain limit of 5% and the gauge resistance is 120 Ω (± 0.3 Ω).
- IP68 rated thermistor sensors (ATC Semitec model IP 68) which are capable of measuring concrete temperature. The thermistors have a temperature range of − 50 to + 110 °C with a 1% tolerance.

**Fig. 5:**
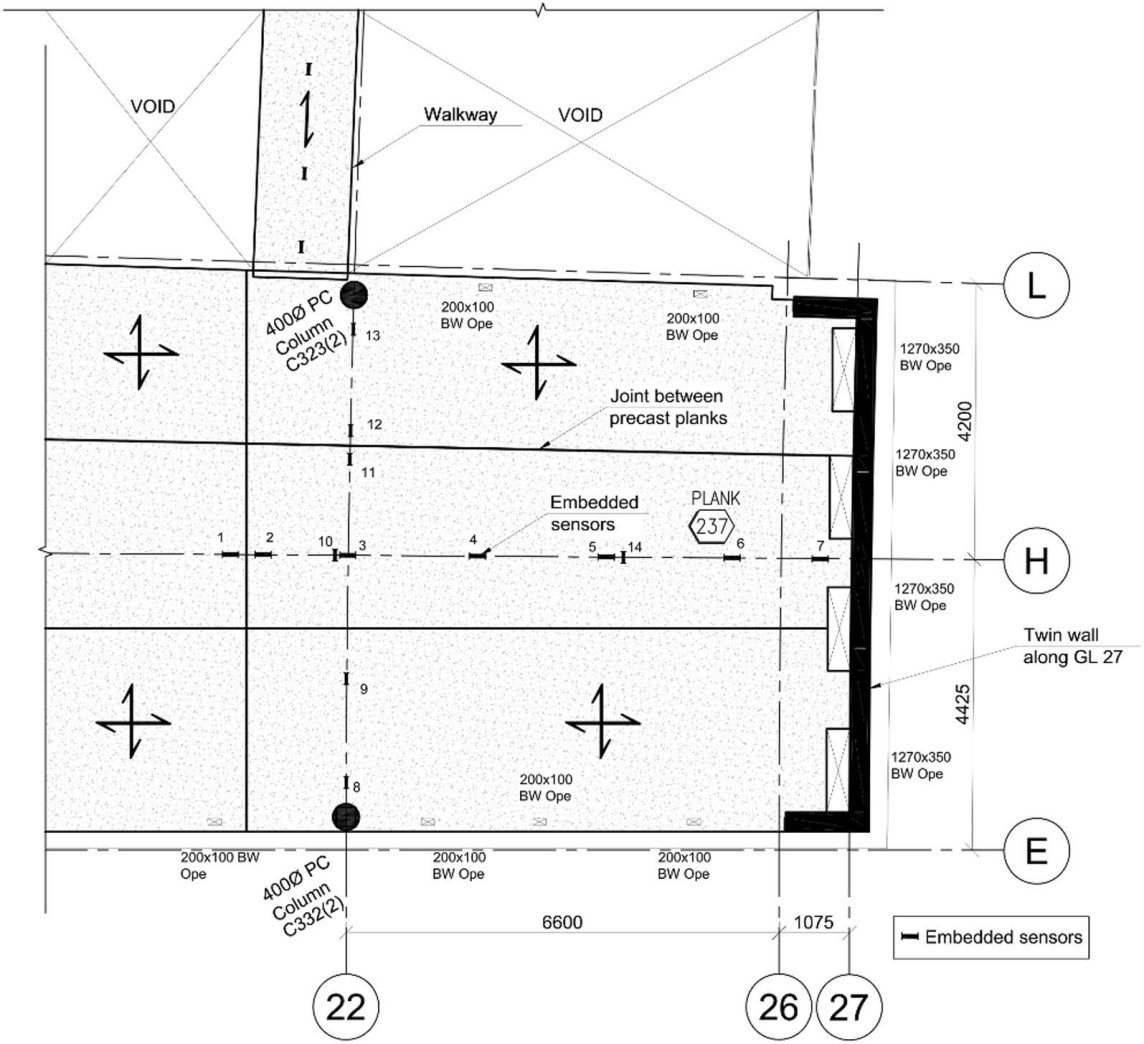
Embedded sensors in one zone of the second floor of HBB (Newell and Goggins 2017).

**Fig. 6:**
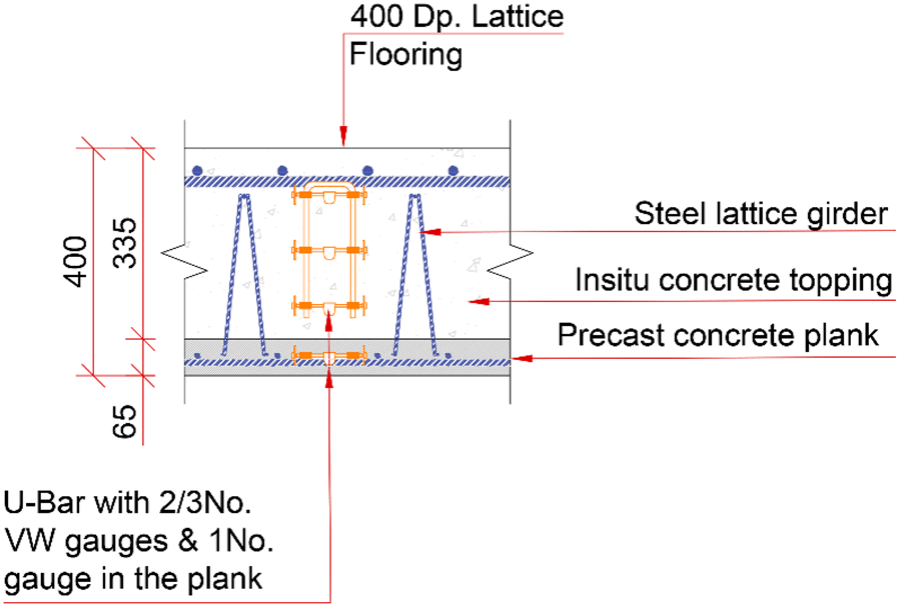
Typical section showing locations of vibrating wire gauge installed in the flat slab system (Newell and Goggins 2017).

The embedded sensors are located within two zones of the second floor of the HBB along a number of orthogonal grids. The location of the embedded sensors in one zone of the second floor slab is shown in Fig. 5. Sensors were installed prior to manufacture of some of the precast lattice girder planks to allow the behaviour of the floor to be monitored during the manufacturing process. The VW gauges are typically arranged such that there are four gauges through the depth of the floor (Fig. 6). This allows the three-dimensional profile of the change in strain and temperature in the floor slab to be monitored.

To collect data from the sensors, a data acquisition system consisting of CR1000 data loggers, AM16/32B multiplexers and AVW200 vibrating wire interface from Campbell Scientific were used to collect and store real-time data from the embedded instrumentation. This system has automatically recorded data from the precast planks since manufacture and the in situ concrete topping since the concrete pour on site. During the construction phase, data was manually downloaded onto a laptop, but after commissioning of the building, data is downloaded over a local network using Campbell Scientific’s NL116 Ethernet interface and compact flash module. This allows long term monitoring of the behaviour of the floor during the operational phase of building. This data is being stored on a dedicated server and is available for students for both teaching and research applications.

### Material Testing

A comprehensive material testing programme was undertaken to measure the properties of the concrete (precast and in situ) in the floor over time. Concrete specimens (cubes, cylinders and prisms) were made and cured in water (in accordance with EN 12390-2 (BSI 2009)) and in air (to replicate environmental conditions on site). The properties of concrete will change over time, and therefore, it is very important to accurately measure the properties which are required to (i) interpret the data from the embedded sensors and (ii) as inputs when modelling the behaviour of the floor. Testing of concrete used in the second floor of HBB was undertaken for 1 year after the in situ topping was poured on site.

### Environmental Conditions

Changes in the environmental conditions surrounding any concrete element will have a significant effect on its behaviour with respect to deformation, strength, durability and service life (Liu et al. 2015; Pour-Ghaz et al. 2009; Sofi et al. 2014). Outdoor weather conditions were provided by the automatic weather station (IRUSE 2017) at the NUI Galway campus which is located less then 1 km from the HBB building. This weather station records air temperature, relative humidity, wind speed and direction, solar irradiance and rainfall at 1 min intervals. As construction proceeded and the erection of the external façade commenced, a standalone data logger (Easy Log EL-USB-2+) which measures ambient temperature and relative humidity was positioned on site in December 2015 so that the precise internal environmental conditions around the second floor were recorded. This is important as the building became weather tight and the commissioning phase of the heating system for the building commenced. In the long term, the internal environment will be monitored using the building management system. When comparing the ambient air temperature readings from the external weather station and internal data logger, the difference is not significant until after August 2016, when the façade of building is fully complete.

### Data Post-Processing

The embedded vibrating wire strain gauges (Gage Technique model TES/5.5/T) measure both change in strain and temperature (via thermistors) in the concrete slab. Changes in the internal temperature of the concrete slab will cause expansion and contraction of both the concrete and vibrating wire gauge that is used to measure change in strain in the concrete. Failure to correct for the thermal effects in the VW strain gauges would lead to erroneous strain readings. The difference between the coefficient of thermal expansion of the concrete and VW strain gauge will give rise to an apparent change in strain in the concrete. The coefficient of thermal expansion of the VW strain gauges is 11με/°C and for this project it was assumed that the coefficient of thermal expansion of the concrete was 9με/°C. The measured strain readings (apparent strain) from the VW gauges are corrected for thermal effects using the following equation so that only strain due to shrinkage, flexure and creep is determined:

1 $$\mu {{\varepsilon }_{\text{load}}}=\mu {{\varepsilon }_{\text{actual}}}-\mu {{\varepsilon }_{\text{thermal}}}=\Delta \varepsilon +\Delta \text{T}\cdot {{\alpha }_{\text{vw}}}-\Delta \text{T}\cdot {{\alpha }_{\text{c}}}$$

where Δε is the change in measured strain; ΔT is the measured change in temperature; α_vw_ is the coefficient of thermal expansion of the VW gauge and α_c_ is the coefficient of thermal expansion of the concrete.

**Fig. 7:**
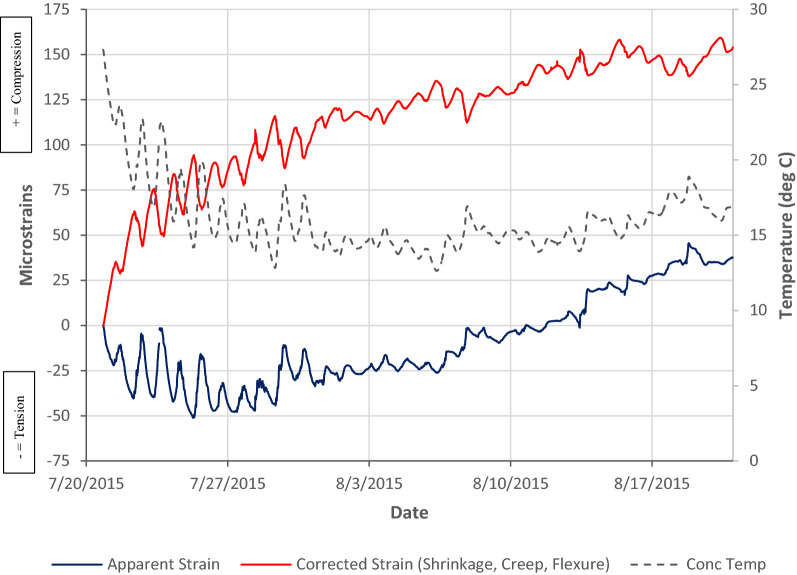
Comparison of apparent and corrected strains from VW strain gauges.

The effect of the thermal correction can be seen in Fig. 7, where the temporal apparent and corrected strains are shown with the slab’s internal temperature for the first month after the in situ topping is poured. Without the temperature correction, the strain data would apparently indicate a tensile strain when the actual strain in the concrete is compressive. The measured strains from the VW strain gauges will record strain due to shrinkage, flexure and creep. Strain due to flexure and creep will not be significant until the supporting props are removed and re-propped. The dominant strain component is shrinkage during the early stages. Further analysis of the measured strains from the embedded sensors and comparison with predicted strains using Eurocode 2 (BSI 2004) is provided in a paper by the same authors focussing on the structural behaviour of the floor during construction (Newell and Goggins 2017).

In the literature, there is no agreement on the time at which the concrete stiffness has exceeded that stiffness of the strain gauge so that measured strains are consistent with the strains in the concrete (Time Zero). Prior to this time, the concrete is not sufficiently stiff to transfer strains to the embedded strain gauge or to restrain the gauge under applied thermal loads. Boulay and Paties (1993) conducted a laboratory and numerical study of the interaction between early-age concrete and VW strain gauges and found that once the concrete modulus exceeded 1500 MPa, the errors between measured and imposed deformations, due to thermal or structural loads were less than 5%.

**Fig. 8:**
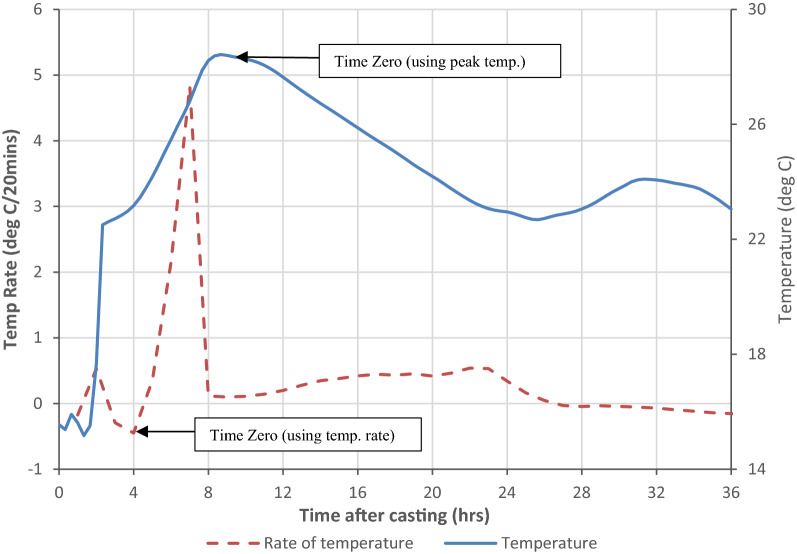
Determination of Time Zero from measured temperature in the concrete.

In this study, the zero point for strain readings is assumed to be time at which the maximum concrete temperature is reached. Prior to the concrete reaching its maximum temperature, the concrete can be assumed to be in, or near to, a zero stress state due to creep (ACI 2002). The in situ concrete topping for second floor slab in HBB in which the sensors were embedded was poured on the 20 July 2015 at 10.00 and the peak concrete temperature was recorded approximately 8–11 h after the pour. Therefore, it is assumed that the zero point is 20.00 on the 20 July 2015. This may be considered conservative as Cusson and Hoogeveen (2007) proposed that that ‘Time Zero’ should be determined when the rate of temperature in the concrete increases sharply. Figure 8 plots the temporal rate of temperature change (left axis) and the measured temperature change (right axis) developing in the concrete after casting from one of the embedded VW strain gauges located in the middle of the slab. This suggests that ‘Time Zero’ occurs somewhere between 4 and 10 h after casting.

### Numerical Model

A one-dimensional finite difference model was developed to model the temperature profile in the hybrid concrete slab. It was assumed that a one-dimensional model through the slab thickness was applicable for the internal sections of the slab and the model is validated against embedded sensors located internally in the slab. The model is based on the numerical method developed by Ross and Bray (1949) and uses heat diffusion theory described by Crank (1975). The model developed is similar to the model described in CIRIA Report C660 (Bamforth 2007) and uses the two component model developed by Dhir et al. (2008) based on test data to predict the heat of hydration. The 400 mm overall thickness of the hybrid slab was modelled with a spatial resolution of 20 mm. At the top surface, four modes of heat transfer are considered: conduction into the concrete, convection, solar radiation (absorption) and thermal irradiation to the surroundings. At the bottom surface, it is assumed that there is no heat transfer due to solar radiation.

The following equations are used in the model to describe heat transfer through the concrete slab:

(i) Conduction:
  2 $${{\text{q}}_{\text{cond}}} = \text{k}\left( {{\text{T}}_{0}} - {{\text{T}}_ 1} \right)/\Delta \text{x}$$
  where k is the thermal conductivity of the concrete, T_0_ and T_1_ are the surface and internal temperature at the first internal node, and Δx is the space increment in the model (through the depth of the concrete).
(ii) Convection:
  3 $${{\text{q}}_{\text{conv}}} = \text{h}\left( {{\text{T}}_{0}} - {{\text{T}}_{\text{a}}} \right)$$
  where h is the convection coefficient (thermal conductance) of the concrete surface (wind speed has a significant effect), T_0_ is the concrete surface temperature, and T_a_ is the ambient temperature.
(iii) Solar radiation:
  4 $${{\text{q}}_{\text{sol}}} = {{\gamma }_{\text{abs}}}{{\text{Q}}_{\text{inc}}}$$
  where γ_abs_ is the solar absorptivity of the concrete and Q_inc_ is the incident solar radiation (measured from a local weather station).
(iv) Thermal irradiation:
  5 $${{q}_{irr}}= \sigma \in \left( \text{T}_{0k}^{4}-\text{T}_{sky}^{4} \right)$$
  where σ is the Stefan–Boltzmann constant, is the emissivity of the concrete, T_0k_ is the concrete surface temperature (in Kelvin), and T_sky_ is the sky temperature.

The difference-calculation remains numerically stable, if the following is valid for the time increment:

6 $$\Delta \text{t}\le \, (\text{C}\rho / 2 \text{k}).\Delta {{\text{x}}^ 2}$$

where Δt is the time increment, Δx is the space increment, C is the specific heat of the concrete, ρ is the density of the concrete and k is the thermal conductivity of the concrete.

At the commencement of the pour, the precast plank is assumed to be the same temperature as the ambient temperature (15.1 °C) and the placing temperature of the concrete is assumed to be 20 °C (5 °C difference is typical based on previous studies in the UK (Anson and Rowlinson 1988; The Concrete Society 2004)). Environmental conditions, measured from the nearby automatic weather station (IRUSE 2017), such as ambient temperature, wind speed and solar irradiation are used as input parameters in the model. The thermal properties (thermal conductivity, specific heat, diffusivity) of the concrete (in situ topping) and precast plank are assumed constant. The specific heat of the concrete (C) in the model was assumed to 1.0 kJ/kg °C and the thermal conductivity of the reinforced concrete (k) was assumed to be 2.34 W/m °C; both values were determined based on the mix design, aggregates (limestone) and percentage reinforcement used in the floor slab in the HBB. The convection coefficient (or thermal conductance) of the surface (h) used in the model at the top and bottom of the slab is based on the measured wind speed (Bentz 2000). When the external cladding was installed, it was assumed that the wind speed was zero for the purposes of determining the convection coefficient and there was no heat transfer due to solar radiation or thermal irradiation.

## Results

The results presented in this paper focus on the manufacture and construction phases of the HBB and relate to a time period of approximately 1 year. The use of sensors to monitor the temperature of concrete during the first few days is common practice, but long-term monitoring is not always implemented. Knowledge of the temperature profile within concrete elements can be used to determine maturity, strength development, when formwork can be removed and potential for early-age thermal cracking. In addition, many sensors are affected by temperature and require temperature correction and, therefore, temperature is a very common parameter in structural monitoring. In this project, the concrete temperature was monitored during the manufacture of the precast plank and pouring of the second floor in situ concrete topping. As outlined previously, the ambient temperature was also monitored during manufacture of the precast plank and during the construction phase of the HBB. The temperature of the concrete slab is being continuously monitored during the operational phase of the HBB using the embedded sensors.

### Early-Age Thermal Effects

Predicting early-age thermal cracking is very complex as there are numerous factors which affect the potential for cracking and properties of the concrete (elastic modulus, compressive strength, tensile strength) are rapidly changing during the early stages of curing. The use of in situ instrumentation in this project allowed the thermal behaviour of the second floor slab to be recorded and this data can be used during the design process for checking early-age thermal cracking. Two important temperature considerations for control of early-age thermal cracking are (Bamforth 2007):

- The temperature rises above the adjoining concrete or substrate and this is applicable to conditions of external restraint. External restraint occurs where an external element restrains the early thermal movements of a member being cast. An external restraint can be classified as a continuous edge restraint, end restraint or intermittent restraint (more than one type of external restraint may apply). The degree of external restraint will depend on the member being cast (wall, slab, beam and their dimensions) and the construction process (supporting elements, reinforcement and construction sequence).
- The maximum temperature differential and thermal gradient within the section. This is the condition of internal restraint and occurs where one part of a concrete pour expands or contracts at a different rate to another part of the same section. It typically occurs in thick sections in which there can be a large temperature differential across the section and can lead to both surface cracking and internal cracking (which may not be observable from the surface). An internal restraint can also occur when there is a large percentage of reinforcement restraining the concrete.

Usually either external or internal restraint dominates; however, there are some situations in which both types of restraint need to be considered.

**Fig. 9:**
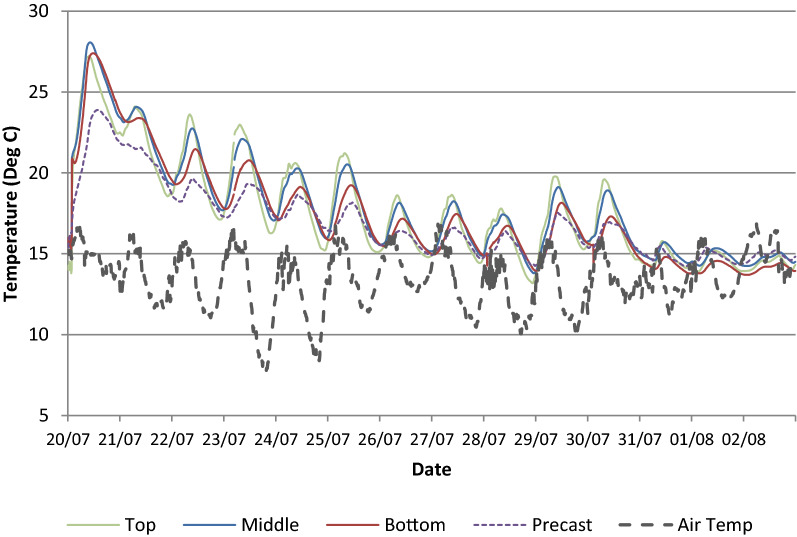
Concrete temperature in the 335 mm in situ structural topping (14 days after pour).

The second floor slab in the HBB consists of a 65 mm thick lattice girder plank and 335 mm in situ concrete topping (400 mm overall thickness). As noted previously, at most locations there were four VW strain gauges positioned through the depth of the slab (top in situ, middle in situ, bottom in situ and precast). The temperature in the concrete at one location in the slab is shown in Fig. 9 for the first 2 weeks after the pour. The heat of hydration effect on the concrete temperatures is clearly observed during the early days of curing. The peak temperature occurs approximately 10 h after pouring when the difference between the concrete temperature and ambient air is approximately 13 °C. Concentrating on the three VW gauges in the in situ structural topping, the ‘middle’ gauge records the highest concrete temperature (28 °C) as it is insulated by the surrounding concrete and is slowest to cool down. As expected, the ‘top’ gauge in the in situ topping records the lowest peak temperature because of heat transfer from/to the external concrete surface to air through convection. The effect of heat transfer through conduction between the in situ topping and the precast plank is also noted as the temperature in the precast plank increases by approximately 9 °C after approximately 14 h since the pour commenced. The relatively low maximum temperature differential (13 °C) between the concrete and ambient air can be partially explained by the mix design for the concrete topping in the HBB. The mix design for the in situ structural topping was 70% CEM I cement and 30% GGBS (ground granulated blast-furnace slag) with total cement content of 330 kg/m^3^. It is common to blend GGBS (typically 30–50%) with CEM I cements to reduce the heat of hydration and likelihood of early-age thermal cracking. CIRIA Report C660 by Bamforth (2007) provides guidance on controlling cracking due to early-age thermal cracking, but it states that direct measurement is the most reliable method for determining the temperature rise during the heat of hydration because of the large number of parameters which can affect its value. The CIRIA document is primarily focussed on construction of walls and does not provide explicit guidance for determining the temperature rise for concrete topping poured on top of a precast plank. The predicted maximum temperature rise using the CIRIA document is approximately 19 °C and this is determined assuming the in situ topping is similar to a suspended floor slab with an uninsulated top surface (the thickness of the wall is increased by a factor of 1.3 and steel formwork is assumed).

The end of the hydration phase is difficult to determine precisely, but after the first few days it appears that the thermal response of the slab is primarily driven by the ambient air temperature and solar gain (Sect. 3.5). After the first day, the maximum and minimum concrete temperatures were recorded in the gauges located near the top of the in situ topping. The peak daily concrete temperatures after the first day decrease through the depth of the slab, the highest in the top and lowest in the precast plank at the bottom of the slab. Similarly, the time lag between the peak ambient temperature and peak concrete temperature increases from top to bottom through the 400 mm thick slab. This illustrates that the top surface of the slab is subject to radiant heating from the sun and the potential of the concrete slab to act as a thermal mass to store and release heat. The time lag between the peak ambient temperature and the peak concrete temperature in the top of the slab in the first few days after the initial heat of hydration varies between 2 and 6 h. It is noted that after the 30 July 2015 (Fig. 9) that the differential between the concrete and ambient air temperature is significantly reduced. This is explained by the fact that the precast planks for the third floor (over the second floor with embedded sensors) were erected on the 31 July 2015 and, therefore, shaded the second floor slab which reduced heat gain from the summer sun.

Large temperature differentials between the surface and centre of concrete elements can lead to internal restraint, which can lead to cracking and this is of critical importance for thick concrete elements. Quantifying and controlling the temperature differential within the concrete section is one of the critical design checks when considering early-age thermal cracking. The maximum recorded temperature differential within the 335 mm thick concrete topping in the second floor slab in HBB never exceeded 3 °C. Using the CIRIA Report C660 (2007), the predicted maximum temperature differential is 13 °C, but this figure is based on applying temperature data for walls and adjusting for suspended floor slab with an uninsulated top surface. The CIRIA report recommends thermal modelling for reliable predictions of thermal behaviour of concrete elements. This paper highlights the advantages of using embedded instrumentation to record the actual thermal behaviour of concrete elements and that it can be used to improve the design process for controlling early-age thermal cracking.

**Fig. 10:**
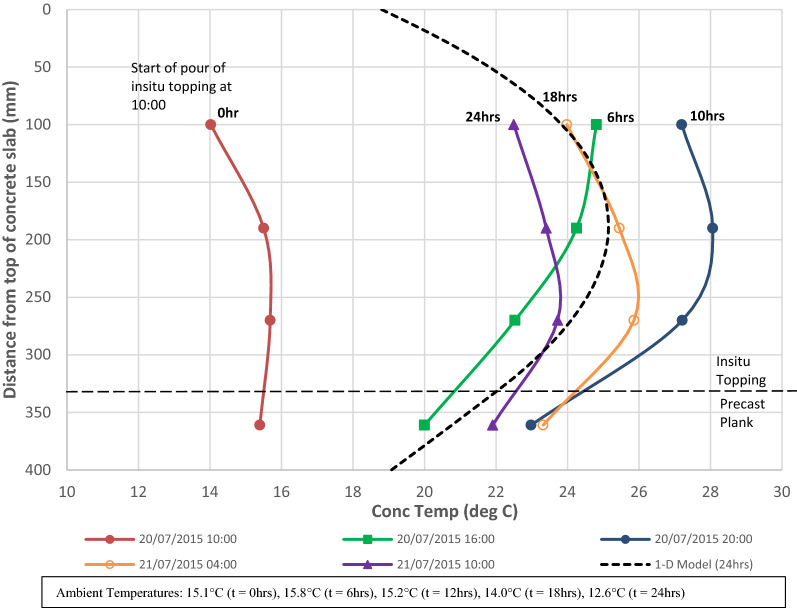
Temperature profile in concrete slab for first 24 h.

The temperature profile through the depth of the slab (400 mm overall thickness) at a location in the middle of the slab is shown for the first 24 h after pouring in Fig. 10. The ambient air temperature at the time of the pour was approximately 15 °C and it is noted that the concrete temperature in the precast plank before the pour of the concrete topping is approximately the same (ambient temperatures shown at bottom of Fig. 10). The peak concrete temperature during the curing phase occurs 10 h after the pour at 20.00 and the highest temperatures are recorded in the middle of the slab. The temperature in the precast plank lags behind the temperatures in the in situ topping and the lowest temperatures in the in situ topping are recorded in the top of the slab as heat is lost to the exposed top surface. During the cooling phase, it is observed that after 24 h the middle of the slab has the highest temperatures as it is the slowest section of the slab to cool down. During the first 24 h, the temperature differentials between the VW gauges never exceeds 3 °C, but it is likely that the maximum differentials between the top surface of the concrete slab and middle of the in situ topping were significantly higher. They are relatively low as the thickness of the in situ topping (335 mm) is relatively thin and allows the heat to dissipate relatively quickly. For mass concrete, a temperature differential limit of 20 °C is often used, but concrete can crack at higher or lower temperature differentials.

As noted previously, in Fig. 9, there are temperature variations through the depth of the slab during the curing phase and at all subsequent stages of construction. Temperatures recorded by the VW strain gauges embedded through the depth of the slab show that they reach different maximum and minimum temperatures and that they occur at different times.

The predicted temperature profile through the slab using the 1-D numerical model after 24 h is also shown in Fig. 10. The model predicts a maximum temperature in the in situ topping of 30 °C at 10 h after the pour commenced and a maximum temperature differential of 8 °C. As noted previously, the maximum measured concrete temperature of 28 °C was recorded after approximately 10 h and the maximum recorded temperature differential was 13 °C. A number of factors could be put forward for the 7% error (~ 2 °C) in predicting the maximum temperature using the 1-D model:

- Similar to other models, the numerical model described in this paper uses adiabatic hydration models (i.e. heat transfer is not lost to/or gained from the surrounding). Previous studies (Vitharana and Sakai 1995; Bamforth 2007) have shown that models based on adiabatic hydration models are more reliable when predicting temperature rises in thick sections rather than thin sections. The in situ topping in HBB was 335 mm thick and would be considered a ‘thin’ section.
- The thermal properties of the concrete (specific heat, thermal conductivity and diffusivity) were assumed to be constant in the model and were based on typical values from the literature. However, the thermal properties of concrete change during the hardening process (DeSchutter and Taerwe 1995) and the accuracy of the temperature prediction is dependent on the thermal properties used in numerical model.
- The numerical model assumes that the in situ topping and hydration process commences at a specific time interval. In practice, the in situ topping was poured on site in layers over approximately 1 hour and therefore may introduce some discrepancies between the predicted and measured temperature in the concrete.
- The accuracy of VW strain gauges for measuring temperature in the concrete is accuracy of ± 1 °C.

### Thermal Gradients

Temperature differentials or thermal gradients between the top and bottom of the slab during the curing phase causes internal restraint in the concrete which may lead to surface or internal cracking (early-age thermal cracks). During the construction phase of the HBB, both the top and bottom of the slab are exposed and, therefore, are affected by daily and seasonal environmental changes. The thermal gradients may lead to slab curvature (curling or warping of the slab) and because typically the slab is subject to some form of external restraint, additional temperature-induced stresses are developed which may create cracks in the concrete. The curvature is upward if the thermal gradient is positive (temperature decreasing from top to bottom) and downward if it is negative. Restraint to slab curvature is the cause of most early-age thermal cracking in pavements (Bamforth 2007).

**Fig. 11:**
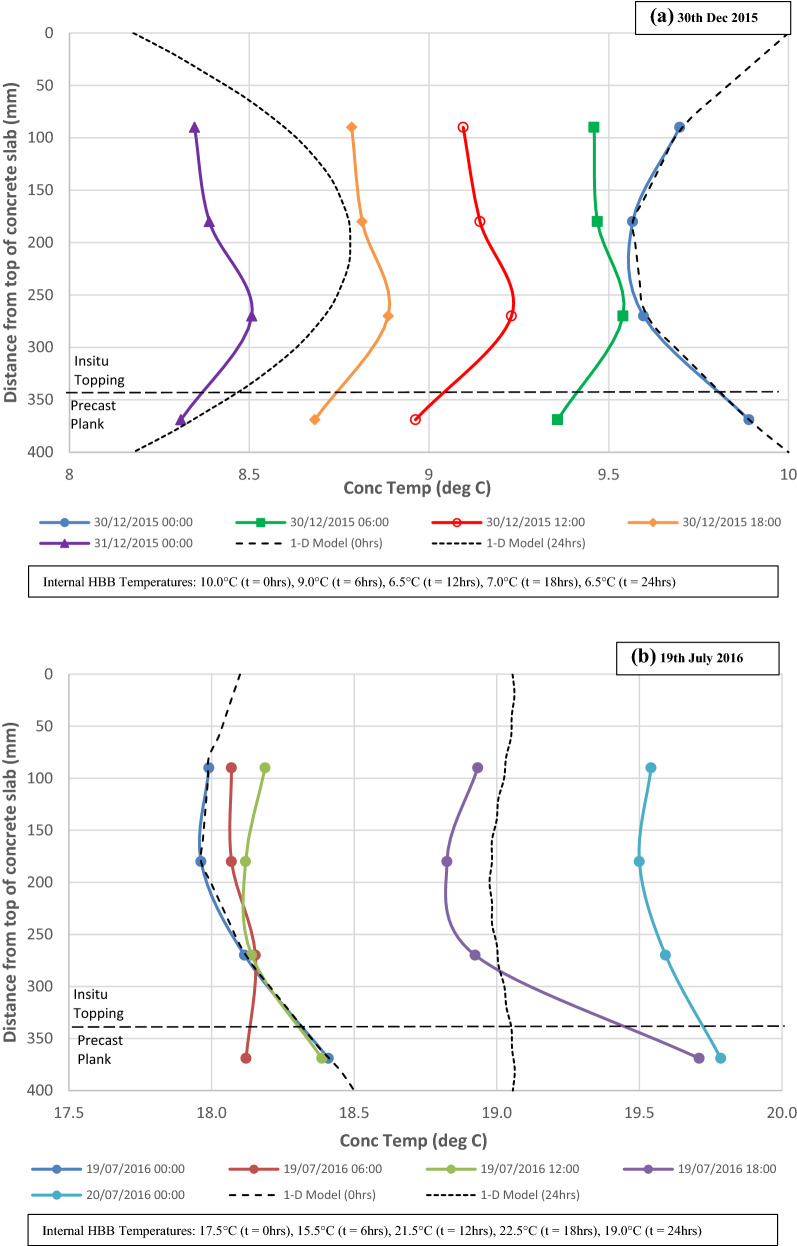
Temperature profile in concrete slab for (**a**) negative temperature differential on 30 Dec 20 15, and (**b**) positive temperature differential on 19 July 2016.

Distinct differences can be observed in the daily thermal gradients in the slab in response to the changes in the ambient air temperature. The thermal gradient in the slab is typically non-linear through the depth of the slab and approximately parabolic in shape at peak differentials. Temperature profiles through the slab are shown in Fig. 11 for 2 days in which there is a negative temperature differential (30 December 2015) and a positive temperature differential (19 July 2016). On the 30 December 2015, the ambient air temperature falls by 5 °C and on the 19 July 2016, the ambient air temperature increases by 15 °C. It can be seen that when the ambient air temperature decreases significantly (typically during night-time in winter months) that the temperature gradient is convex as both the top and bottom exposed surfaces of the slab cool quicker than the inner core of the slab. On the 30 December, the thermal gradient changes from convex to concave as the ambient air temperature decreases and the temperature of exposed surfaces of the slab become lower than the inner core of the slab. Conversely, when the ambient air temperature increases significantly (typically during day-time in summer months) the temperature gradient is concave as both the top and bottom exposed surfaces of the slab heat up quicker than the inner core of the slab. The recorded thermal gradients in the slab are rarely linear or uniform during the construction phase as the temperature of the slab is continuously changing in response to the diurnal temperature cycle.

The predicted temperature profiles on the 30 December 2015 and 19 July 2016 using the 1-D model are also shown in Fig. 11 (a, b respectively). The input temperatures for the concrete for the initial time step on each day were based on the temperatures measured by the four embedded sensors in the slab. The ambient temperature was based on the internal temperature in the building measured by a sensor that was positioned adjacent to the slab when the external cladding commenced (Sect. 3.3). The predicted temperatures by the model indicate similar developments to the measured temperatures and in both cases the predicted temperature are within 1 °C of the measured temperatures after 24 h (note: accuracy of the VW gauges is ± 1 °C). Similar to the measured profiles, the predicted temperature profile changes from convex to concave on the 30 Dec 2015 over the duration of the day as the ambient temperature decreases.

Although the 1-D finite-difference numerical model described in this paper is relatively simple, it can be used to predict the temperature profile during the early-age heat of hydration phase and at later stages when the environmental conditions are the dominant factor on the temperature in the concrete slab. During curing of the concrete slab, the model will tend to predict temperature rises which are conservative, but could be used to identify potential early-age thermal cracking. Accurate prediction of the concrete temperatures in slabs is critical if the thermal mass of the concrete is used as one of the passive design strategies to control comfort (heating and cooling) in a sustainable manner. Exploiting the potential of exposed concrete in buildings can lead to enhanced energy efficiency and carbon savings over the lifetime of the building.

### Horizontal Spatial Temperature Distribution

**Fig. 12:**
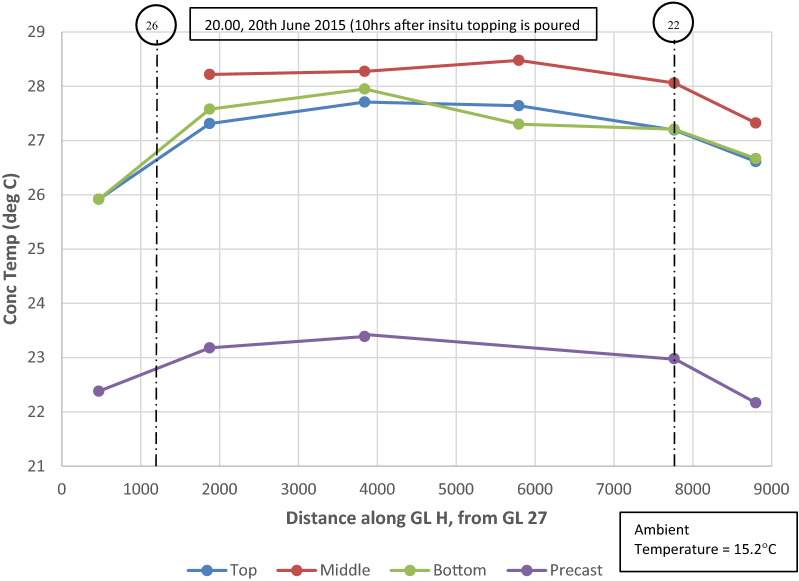
Temperature distribution in concrete slab along GL H at approximately peak temperature during hydration phase (20.00, 20 June 2015).

Thermal gradients were also recorded horizontally in the slab in addition to vertically through the depth of the slab as outlined above. The temperature distribution along gridline (GL) H (refer to Fig. 5) recorded by VW gauges located in the top, middle and bottom of the structural topping and in the precast plank are shown in Fig. 12, 10 h after the in situ topping is poured (10.00 20 June 2015). This time period (20.00) is approximately the peak temperature in the concrete slab during the hydration phase. The lag in temperature increase in the precast plank is clearly shown after the in situ topping is poured and as noted previously, the maximum temperatures in the concrete slab are recorded in the middle of the slab.

**Fig. 13:**
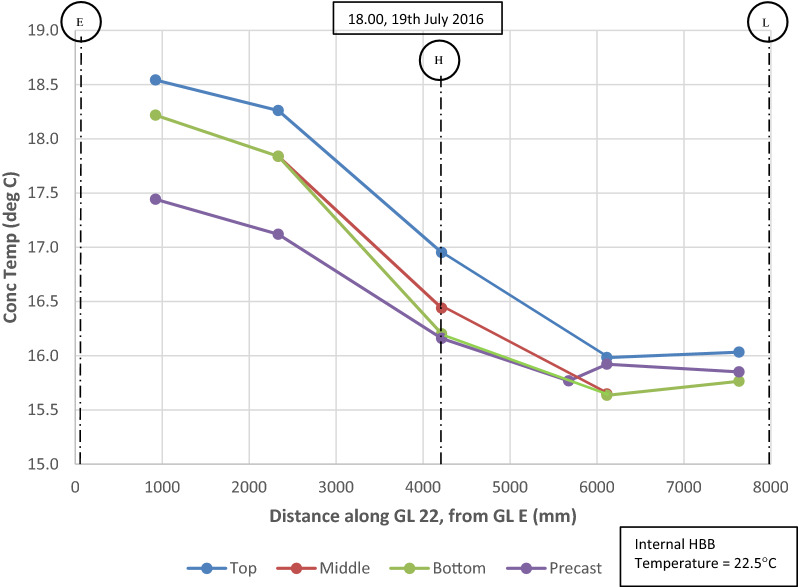
Temperature distribution in concrete slab at 18.00, 19 July 2016 along GL 22.

After the curing phase, the horizontal temperature distributions in the instrumented section of the second floor concrete slab are relatively uniform and do not exceed 3 °C difference between sensors along GL H or GL 22 (embedded sensors positioned along approximately 9 m length of slab in orthogonal directions). However, local deviations in the horizontal temperature distributions are noted in the slab perimeter along GL 22. The horizontal temperature distributions for the slab along GL 22 are shown in Fig. 13 for the 19 July 2016 (18.00), which corresponds to the highest recorded daily ambient temperature during the construction phase. There is a noticeable decrease in the slab temperature along GL 22 and this is typical of the horizontal temperature distribution on days of high ambient temperature during the construction phase. On the 19 July 2016, there is a recorded temperature differential of 3 °C across the width of the slab (total width along GL 22 = 8.5 m from GL E to L) which equates to a horizontal temperature gradient of 0.37 °C/m. The slab perimeter along GL E is on the South-West external elevation of the building and the slab perimeter along GL L is internal (adjacent to atrium). It is assumed that the higher temperatures recorded in the slab perimeter adjacent to GL E are due to solar gain during the day (Sect. 3.5 provides further analysis of solar irradiance) as there is continuous glazing along GL E. This suggests that the orientation of structural elements and exposure conditions can have a significant effect on the spatial temperature distribution in concrete slabs, particularly in regions which may be subject to significant solar radiation.

### Coefficient of Thermal Expansion

The coefficient of thermal expansion of concrete (α_c_) is a measure of the free strain (unrestrained concrete) produced by a unit change in temperature and its magnitude will control the expansion and contraction of the concrete in response to a given temperature change. In design of concrete structures, the value of α_c_ can influence the control of early-age thermal cracking, provision of movement joints and construction tolerances for elements connected to concrete. A concrete with a low coefficient of thermal expansion is desirable when trying to control the risk of early-age thermal cracking; the type of aggregate used in the concrete is the dominant factor with respect to the coefficient of thermal expansion of concrete. The value of α_c_ can vary from 8 to 13με/°C depending on the type of aggregate used in the mix. Eurocode 2 (BSI (British Standards Institution) 2004) recommends a value of 10με/°C for normal weight concretes if no information is available. In the HBB project, limestone aggregates were used for the concrete for both the lattice girder precast plank and the in situ structural topping and, therefore, a value of 9με/°C was assumed for the concrete based recommendations by Bamforth et al. (2008). It should also be noted that α_c_ changes over time and with moisture content.

**Fig. 14:**
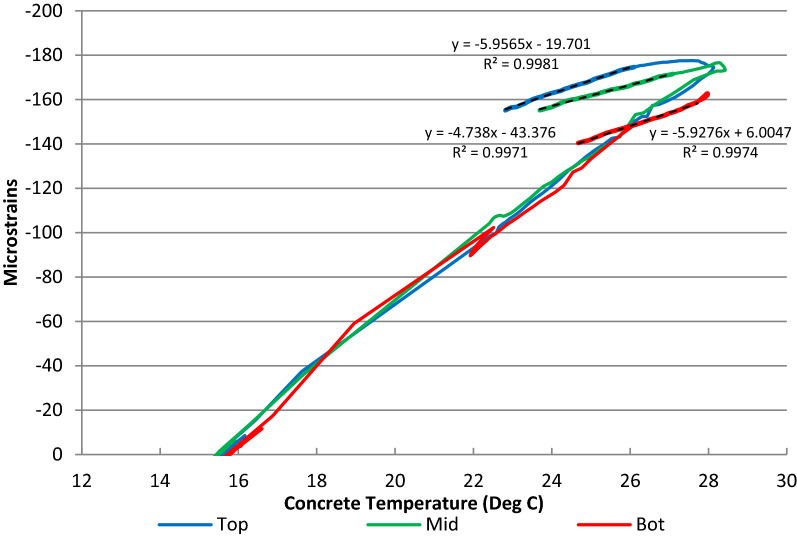
In situ strain–temperature curve for the in situ topping at one location in the slab.

There is no standard method for determining the coefficient of thermal expansion of concrete in CEN, although there is a method provided in BS EN 1770 (BSI 1998) related to repair materials. For large projects, particularly when thermal cracking is critical, it is advisable to accurately determine the in situ thermal properties of the concrete so that the design is based on actual concrete properties rather than assumed behaviour. The embedded VW strain gauges in the concrete floor in the HBB allow the in situ restrained coefficient of thermal expansion of concrete (α_r_) to be measured for the in situ topping that is poured on top of the precast plank. The slope of the in situ strain–temperature relationship during the cooling phase of the temperature cycle can be used to determine α_r_ at a number of locations in the slab and at different depths in the slab. In Fig. 14, the strain–temperature curves are plotted for three VW strain gauges in the top, middle and bottom at one location in the in situ topping. It is observed that α_r_ is lower in the middle (4.7με/°C) of the concrete in comparison with the top (6.0με/°C) and bottom (5.9με/°C) and this finding was consistent at all locations in the slab.

**Fig. 15:**
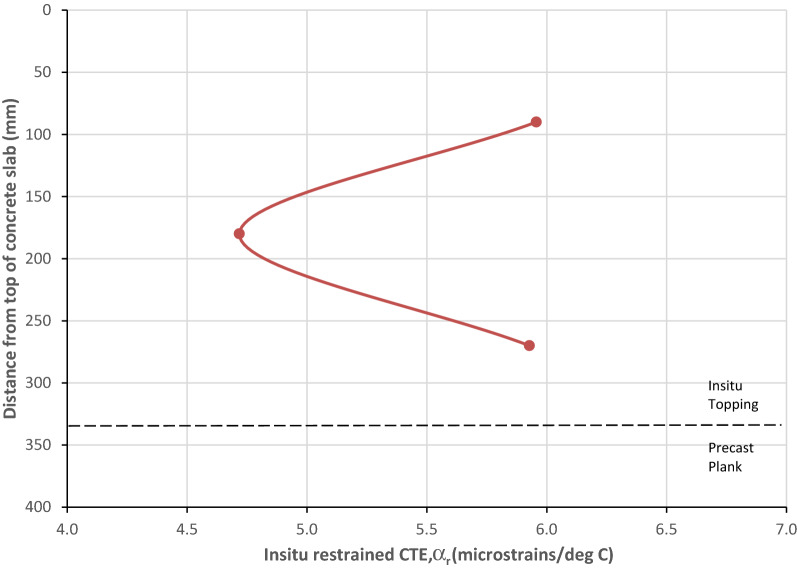
Variation of in situ restrained coefficient of thermal expansion in slab.

The variation of α_r_ through the depth of the slab is shown in Fig. 15 based on the values of α_r_ determined in strain–temperature plot previously (Fig. 14). The average in situ restrained coefficient of thermal expansion of concrete (α_r_) using the embedded sensors in the 335 mm thick in situ topping was 6.6με/°C with a coefficient of variation (Cv) of 13% (max = 8.1με/°C, min = 4.7με/°C). The average α_r_ for gauges located in the middle of the in situ topping was 5.7με/°C (Cv = 11%) and the average α_r_ for gauges located in the top and bottom of the in situ topping was 6.9με/°C (Cv = 10%).

In previous experimental studies on mass concrete foundations (Bamforth 1980), the variation of α_r_ with depth of pour was found to be approximately represented by a parabolic curve and this correlates with the in situ data from the slab in the HBB even though the top of the in situ topping is exposed and the bottom of the in situ topping is partially insulated by the precast plank. The variation of α_r_ with depth correlates with the variation in concrete temperature, the central core being subjected to the greater temperature rise and imposing movement on the cooler surface regions. The reduced value of the restrained coefficient of thermal expansion of concrete (α_r_) in the middle of the in situ topping in comparison with the top and bottom (Fig. 15) indicates that the central core of the slab is subject to more restraint during the cooling phase. As the core of the concrete cools, its thermal contraction is restrained by the already cooler external sections of the in situ topping and the potential for internal cracking. Early-age thermal cracking will occur in the concrete if the restrained strain exceeds the tensile strain capacity of the concrete.

Restraint factors (R) are used in many design codes to quantify the degree of restraint when two concrete elements are cast against each other (R = 0.0 unrestrained; R = 1.0 full restraint). Annex L of Eurocode 2 (BSI 2006) provides guidance for determining restraint factors for common construction situations, but there is little guidance on suspended slabs; however, BS 8110 (BSI 1985) (superseded standard) suggested a restraint factor of between 0.2 and 0.4 should be used for suspended slabs. There is no information on restraint factors for hybrid concrete slabs such as precast concrete lattice girder flat system used in HBB. Incorrect values of restraint factors used in design can lead to inefficient over-design or under-design with potential cracking. Therefore, accurate estimation of restraint factors is critical for design and control of early-age thermal cracking.

CIRIA Report C660 (Bamforth 2007) outlines how the restraint factors can be determined using measured in situ data. The restraint factor (R) can be calculated using:

7 $$R= \frac{\left( {{\alpha }_{c}}- {{\alpha }_{r}} \right)}{{{\alpha }_{c}}}$$

where α_c_ is the coefficient of thermal expansion of unrestrained concrete (free strain) and α_r_ is the in situ restrained coefficient of expansion strain which can be measured as outlined above. Assuming that α_c_ was 9με/°C for the in situ topping in the HBB using limestone aggregates, values for the restraint factors can be determined using the in situ values for restrained coefficient of expansion strain (α_r_). The average restraint factor at the bottom of the in situ topping which is cast on top of the precast plank was 0.25 (Cv = 34%). A method for estimating the restraint factor at a joint (R_j_) was proposed by ACI (American Concrete Institute) (2002):

8 $${{R}_{j}}= \frac{1}{1+ \frac{{{A}_{n}}}{{{A}_{o}}} \frac{{{E}_{n}}}{{{E}_{o}}}}$$

where: *A*_*n*_ is the cross-sectional area of the new (restrained) pour; *A*_*o*_ is the cross-sectional area of the old (restraining) concrete; *E*_*n*_ is the modulus of elasticity of the new pour concrete; *E*_*o*_ is the modulus of elasticity of the old concrete.

It is recommended that for a slab cast against an existing slab that the relative area (A_n_/A_o_) is in proportion to the relative thicknesses of the two slabs (h_n_/h_o_). Using the above equation, the estimated restraint at the interface of the in situ topping and the precast plank after 12 h is calculated as R_j_ = 0.24. This compares favourably with the restraint factor determined using the in situ data (R = 0.25) from the embedded instrumentation during the cooling phase after the heat of hydration even though there is a large variability. Thus, the equation developed by the ACI could be a useful method for determining restraint factors in hybrid concrete slabs.

### Solar Irradiance

In addition to consideration of ambient air temperature, the effect of solar radiation on the behaviour of concrete floors must be considered, particularly in regions in which exposed concrete may be subject to high levels of solar radiation during construction and/or in service. Better understanding and prediction of the thermal behaviour of concrete slabs and their ability to store and release heat is also very important with respect to better design of buildings with exposed concrete surfaces (using thermal mass).

The automatic weather station (IRUSE 2017) at the NUI Galway campus measures both total and diffuse solar irradiance (W/m^2^) and this allowed the daily total solar irradiance (kJ/m^2^) to be determined and compared with the thermal behaviour of the precast plank and hybrid concrete lattice girder flat slab during construction. The total area of the second floor slab which was instrumented had limited exposure to solar radiation during construction because 11 days after the in situ topping was poured (31 July 2015), precast planks for the third floor were erected. The third floor precast planks located directly over the second floor provided shading for the majority of the slab, except at the perimeter which was still subject to partial solar radiation.

**Fig. 16:**
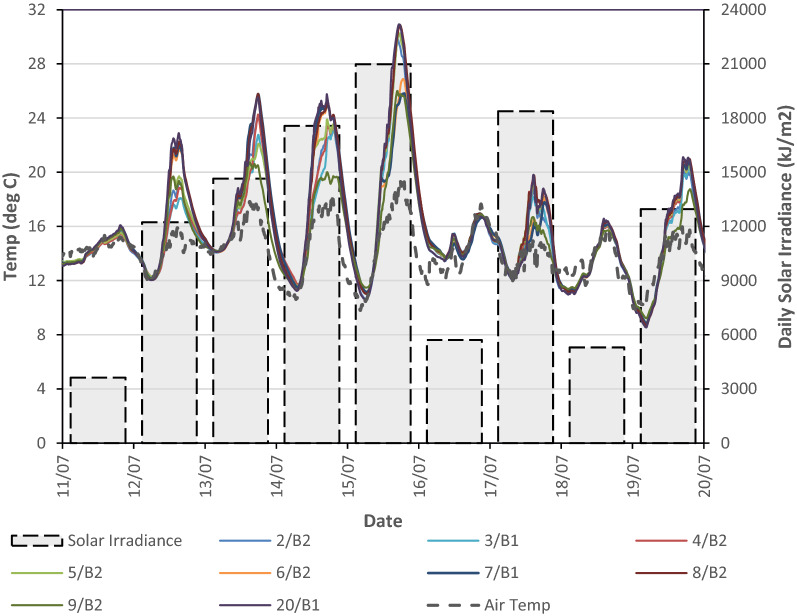
Comparison of concrete temperature and solar irradiance in the precast plank.

The 65 mm thick precast planks for the second floor were erected on the 6 July 2015 and data was recorded after the 10 July 2015. Nine VW strain gauges were embedded in one of the 65 mm thick precast planks (Plank 237, Fig. 5) and this allowed the thermal behaviour of the precast plank to be analysed for the 9 days prior to pouring the in situ topping on the 20 July 2015. The concrete temperature in plank 237 and the daily solar irradiance is shown in Fig. 16 for the 9 days prior to the pour of the in situ topping. On the days of low solar irradiance (11 July, 16 July and 18 July), the concrete temperature and ambient air temperature are closely aligned and the maximum difference between the concrete temperature and ambient air temperature is less than 2°. On these days, there is very good correlation between the diurnal temperature cycle and concrete temperature because the plank is relatively thin and there is little solar radiation.

This contrasts with days of high solar irradiance, when the concrete temperature in the plank is significantly greater than the ambient temperature. On the 15 July, which had the highest solar irradiance prior to pouring the in situ topping, the temperature in the plank was almost 12° higher than the ambient temperature. This highlights the potential for generation of significant induced thermal strains in concrete elements during construction, particularly in regions of high solar radiation. At night-time, the precast plank returns to ambient temperature and this illustrates the effectiveness of concrete with respect to exchanging heat with the environment (admittance) and potential of using concrete to manage thermal energy flows in a building.

**Fig. 17:**
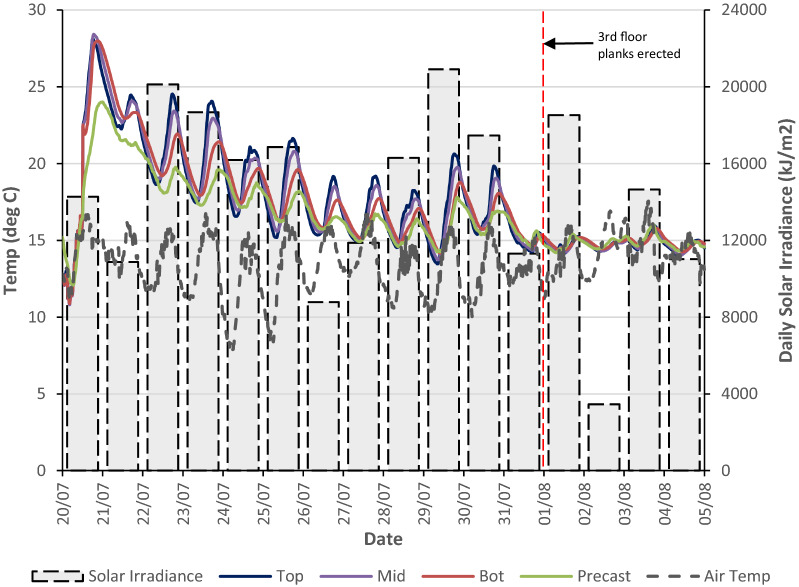
Comparison of concrete temperature and solar irradiance in the in situ topping.

The concrete temperature in the in situ topping in the centre of the slab and the daily solar irradiance is shown in Fig. 17 for the 16 days after the pour of the in situ topping (20 July–4 August 2015). The exposed surface of the in situ topping of the floor slab was not covered during any stage of the construction. This is typical of concrete slabs poured during the summer in Ireland and the UK if high temperatures or strong winds are not anticipated. The impact on solar heat gain can be observed on the 22 July 2015 (3 days after the pour) when the peak temperature in the top of the slab exceeds the peak temperature of the previous day (21 July 2015) even though the ambient temperature was broadly similar for the 2 days. As the heat of hydration dissipates, it would be expected that the concrete temperatures would reduce at this stage and correlate with the ambient temperature; however, the high solar irradiance on the 22 July 2015 increases the concrete temperature in the top of the floor slab. As expected, because the top of the slab is directly exposed to the sun, the temperatures through the depth of the slab decrease from top to bottom and the lowest concrete temperatures are recorded in the precast plank at the bottom of the slab. The highest daily solar irradiance is recorded on the 29 July 2015 and there is a noticeable increase in concrete temperatures on this day. The peak concrete temperature of 21 °C is recorded in the top VW gauge (90 mm below top surface of the slab) at 18:20 and this is approximately 6 °C higher than the ambient temperature (15 °C). At this time, the temperature in the precast plank is almost 4 °C lower and this represents a vertical temperature gradient of 0.13 °C/cm through the depth of the slab. The significant shading effect of the third floor precast planks after the 31 July 2015 is very noticeable as after this date, the concrete temperature stabilises through the depth of the slab and temperature does not exceed the ambient temperature even though there was high solar irradiance on the 1 and 3 August 2015.

### Thermal Loadings

The magnitude and source of thermal induced loadings will vary during the lifetime of concrete structures. Initially, the heat generated during the heat of hydration phase can cause strains which may result in cracks which will affect the durability and performance of concrete elements. After the curing phase, interaction of the concrete with the environment (ambient temperature and solar radiation) can lead to daily and seasonal temperature changes within the structure which will cause the material to expand and contract. If movement in the structure is restrained, stresses may be induced which may contribute to cracking in the structure. In some cases, the thermal induced stresses may exceed the stresses induced by service loads (permanent and variable loading).

An estimate of the thermal strains induced in the concrete slab may be determined by assuming a coefficient of thermal expansion of the 9με/°C for the concrete. As noted in Sect. 3.1, the measured peak temperature during concrete during curing was 13 °C and, therefore, this equates to thermal strains of approximately 117με in the in situ topping. Cracking will occur in the concrete if the tensile strain capacity of the concrete at that time is less than the strains induced by the temperature change during the hydration phase. After the initial curing period (first 7 days), cracking due to early-age thermal cracking is unlikely as the tensile strain capacity of the concrete increases over time.

Exposed concrete surfaces are subject to temperature increases due to solar radiation which can induce curvature in the slab due to the creation of temperature differentials through the concrete (Sect. 3.5) and also increase the average temperature of the whole section which will increase the linear expansion in comparison to expansion due to ambient temperature alone (concrete in the shade). Thermal induced stresses due to solar radiation are particularly important for structures with exposed concrete such as multi-storey car parks, bridges and reservoirs. The embedded sensors in the slab of the HBB indicate that consideration of solar radiation during construction may also need to be considered during design in regions of high levels of sunshine.

## Conclusions

A detailed account of a real-time monitoring programme is described which allows the thermal behaviour of a hybrid concrete floor during the construction phase to be investigated. The project involved embedding sensors in the precast concrete plank during manufacture and the instu topping during construction so that the response of a hybrid concrete floor to temperature variations for 1 year could be monitored. The relationship between concrete floor and external environmental conditions was investigated using data from an automatic weather station located close to the building.

The data from the embedded sensors demonstrates that thermal behaviour of the concrete changes during the construction process. For the first few days after the in situ topping is poured, the heat of hydration is the dominant factor and has the potential to cause thermal cracks if the tensile strains generated in the concrete exceed the tensile strain capacity of the concrete. Although the concrete slab monitored in this project was exposed to direct solar radiation for a short period of the time, the significant effect of solar radiation on the concrete temperatures in the slab were recorded. High levels of solar radiation have the potential to cause curvature of the slab and also increase the average temperature of the concrete section. During the monitoring period described in this paper (1 year), differences in the thermal response of the slab to diurnal and seasonal temperature variations were observed and time lag between ambient temperature and concrete temperatures were noted. Variations in concrete temperatures in the concrete slab were recorded vertically through the depth of the slab and horizontally in the region of the slab which was monitored.

A one-dimensional finite-difference model is described which takes account of the various heat transfer components acting on the slab and allows prediction of the temperature profile in the slab during construction and operational phase of the building. Although the model is conservative when predicting the temperature rise during the heat of hydration, it can be used to model the temperature rise and temperature differential in a hybrid concrete floor when the in situ structural topping is poured. The thermal response of the floor to the diurnal and seasonal changes in the ambient temperature is also modelled using the numerical model.

This paper demonstrates the important role which embedded sensors can play for better understanding and prediction of thermal behaviour of concrete components in real buildings. The comprehensive thermal information from the embedded sensors described in this paper have many potential applications for designers of concrete buildings:

- The data presented in this paper can be used in conjunction with laboratory experiments to develop and validate numerical models which will allow designers to simulate the thermal behaviour of concrete components. This is particularly important in buildings in which the thermal mass of exposed concrete is used to regulate the internal environment and reduce energy consumption.
- Although, hybrid concrete construction is increasingly popular in concrete structures, there is little data available on the actual thermal behaviour of concrete components. The data presented in this paper can be used in conjunction with laboratory experiments to develop and validate numerical models which will allow designers to simulate the thermal behaviour of concrete components. This is particularly important in buildings in which the thermal mass of exposed concrete is used to regulate the internal environment and reduce energy consumption.
- The effect of solar gain on exposed concrete components can be significant in regions which have high levels of solar radiation and needs to be considered at design stage. Thermal strains induced by solar irradiance can induce additional curvature and moments in slabs. The thermal data presented in this paper is related to actual weather data and can be used for analysing the effect of solar irradiance on concrete slabs.
- Guidelines for designers in codes of practice in relation to restraint for suspended slabs is limited. At design stage, it is very difficult to determine the degree of restraint and the nature of restraint (internal, external or a combination of both). The methodology described in this paper for determining the restraint factors based on the measured restrained coefficient of thermal expansion is applicable to similar hybrid precast concrete slabs.

Thermal effects during the construction and operational phases of concrete, if not considered during the design process, can have a significant negative effect on the performance of concrete structures. As performance requirements of buildings continue to increase, designers must adopt a holistic approach to design and be able to demonstrate how the various components and materials work in unison to enhance the overall performance of the building. If implementing a passive design strategy, designers will require accurate numerical models validated using field data from buildings to predict the thermal behaviour of concrete floors so that it can be optimised with respect to controlling the internal environment, thermal mass and energy efficiency.
